# Recurrent Optic Neuritis as a Misleading Presentation of Leber Hereditary Optic Neuropathy: The Need for High Clinical Suspicion in Young Men

**DOI:** 10.7759/cureus.81863

**Published:** 2025-04-08

**Authors:** Nurul Husna Azmi, Anhar Hafiz Silim, Rajasudha Sawri Rajan, Rona Asnida Nasaruddin

**Affiliations:** 1 Ophthalmology, Hospital Selayang, Selayang, MYS; 2 Ophthalmology, Hospital Universiti Kebangsaan Malaysia, Kuala Lumpur, MYS

**Keywords:** leber hereditary optic neuropathy (lhon), mitochondrial disorder, mitochondrial dna mutations, optic neuropathy, recurrent optic neuritis

## Abstract

A 29-year-old Chinese gentleman presented with acute-onset right eye (RE) central scotoma and blurring of vision. Upon presentation, RE visual acuity (VA) was 6/30. The RE optic disc (OD) was mildly swollen, but other findings were unremarkable. A computed tomography (CT) imaging study showed no evidence of a space-occupying lesion. The erythrocyte sedimentation rate (ESR) and other laboratory blood results were normal. The patient was empirically treated with a course of steroids for optic neuritis (ON), but no marked improvement was noticed. He presented again two months later with worsening visual problems in both eyes (BE). The right and left VA reduced to 6/36 and 6/18, respectively. BE OD appeared swollen and hyperemic. BE central scotoma was confirmed with the Humphrey Visual Field (HVF) test. A magnetic resonance imaging (MRI) study was conducted and only revealed a mild heterogenous hyperintensity of the right optic nerve. There is no other evidence of central nervous lesion suggestive of demyelinating disease. A blood investigation for Leber hereditary optic neuropathy (LHON) genetic testing was done, and a confirmatory result of mitochondrial DNA (mtDNA) G11778A pathogenic mutation was detected.

## Introduction

Leber hereditary optic neuropathy (LHON) is a rare mitochondrial disorder predominantly affecting young men, characterized by progressive vision loss due to optic neuropathy [1]. This condition follows maternal inheritance, with approximately 95% of cases linked to one of three mitochondrial DNA (mtDNA) point mutations m.3460G>A, m.11778G>A, and m.14484T>C. These mutations impair electron transfer complex I function, ultimately triggering apoptotic retinal ganglion cell (RGC) death [2].

LHON is recognized as one of the most common mitochondrial diseases, with reported prevalence rates ranging from one in 27,000 in Northeast England to one in 45,000 across European populations. The disorder demonstrates a notable male predominance (80%-90%), with the typical age of onset between 15 and 35 years [3]. However, unlike other mitochondrial disorders, LHON's pathogenesis is not solely attributed to genetic mutations. Environmental and epigenetic factors, including alcohol and tobacco use, nutritional deficiencies, metabolic disturbances, toxins, certain medications, and psychological stress, have been implicated as potential triggers [4,5].

Clinically, LHON often presents as painless, subacute central vision loss in one eye, with the contralateral eye becoming affected within weeks to months. In approximately 25% of cases, bilateral onset occurs simultaneously. During the acute phase, common fundoscopic findings include optic disc (OD) hyperemia, peripapillary telangiectatic vessels, vascular tortuosity, and retinal nerve fiber layer (RNFL) pseudo-edema. These features may gradually progress toward optic atrophy and permanent visual impairment [4-6].

In this report, we discuss a unique case of LHON initially misdiagnosed as recurrent optic neuritis, underscoring the importance of clinical suspicion in young male patients presenting with unexplained optic neuropathy.

This article was previously presented as an E-Poster at the 3rd MOH Annual Glaucoma Symposium (MAGS) on March 4-5, 2022.

## Case presentation

A 29-year-old Chinese man with no prior medical conditions presented with acute-onset right eye (RE) central scotoma. The patient denied experiencing eye pain, Uhthoff's phenomenon, Lhermitte's sign, or any viral-like illness prior to symptom onset. He also denied any history of similar episodes. There was also no family history of similar presentations or blindness. Upon presentation to our clinic, RE visual acuity (VA) was 6/30 with positive reverse afferent pupillary defect (RAPD), whereas left eye (LE) vision was 6/6. The right eye (RE) exhibited reduced optic nerve function on testing. Fundoscopic examination revealed mild optic disc (OD) swelling; however, no macular star or telangiectatic vessels were observed. The left eye examination was normal. On systemic examination, the patient was of medium build with normal blood pressure, and no other abnormalities were noted. A computed tomography (CT) scan showed no evidence of a space-occupying lesion, although it did not suggest features of a demyelinating process. The erythrocyte sedimentation rate (ESR) and other laboratory blood results were normal. The patient was treated for atypical optic neuritis and initiated on a steroid regimen, receiving intravenous methylprednisolone 250 mg four times daily for three days, followed by oral prednisolone at 1 mg/kg once daily for 11 days. Despite completing the steroid treatment, there was no marked improvement in vision or optic nerve function test.

**Figure 1:**
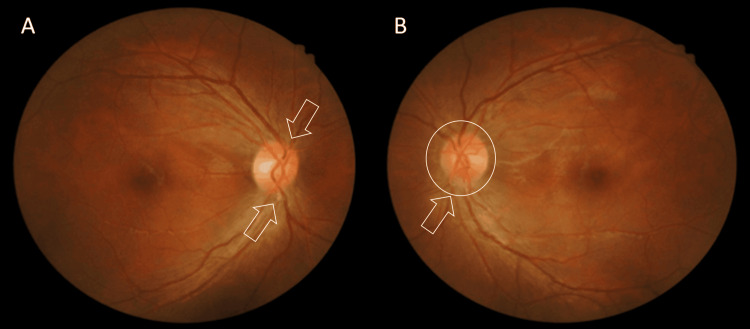
Bilateral fundus photo (A) The right optic disc appeared hyperemic, with swelling noted in both the superior and inferior regions (arrows). (B) The left optic disc appeared circumferentially swollen and hyperemic (arrow).

Two months later, he presented again with worsening visual symptoms in both eyes (BE). His visual acuity (VA) had deteriorated further, with the right eye (RE) reduced to 6/36 and the left eye (LE) to 6/18. Fundus examination revealed optic disc swelling in both eyes (BE). The right optic disc appeared hyperemic, with swelling observed in both the superior and inferior regions, whereas the left optic disc was circumferentially swollen and hyperemic (Figure 1).

**Figure 2:**
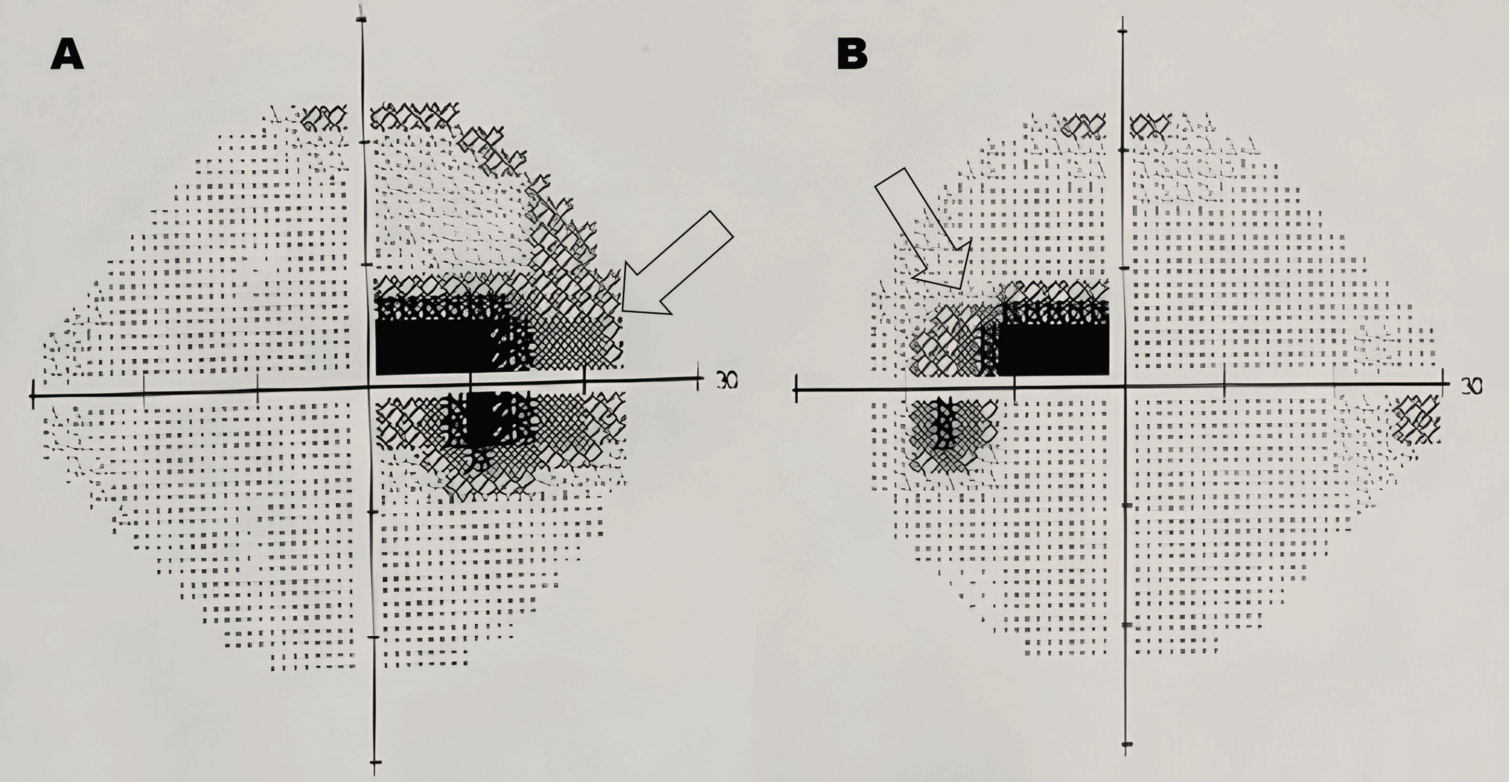
HVF test of bilateral eyes (darkened areas indicate scotomas (areas of reduced or lost vision)) (A) Right eye dense central scotoma extending both superiorly and inferiorly (arrow). (B) Left eye central scotoma, slightly less dense compared to the right eye (arrow). HVF: Humphrey Visual Field

The Humphrey Visual Field (HVF) test was performed, which revealed bilateral central scotomas, characterized by a marked reduction in sensitivity within the central 10-15 degrees of the visual field in both eyes, while the peripheral fields remain largely intact (Figure 2).

**Figure 3:**
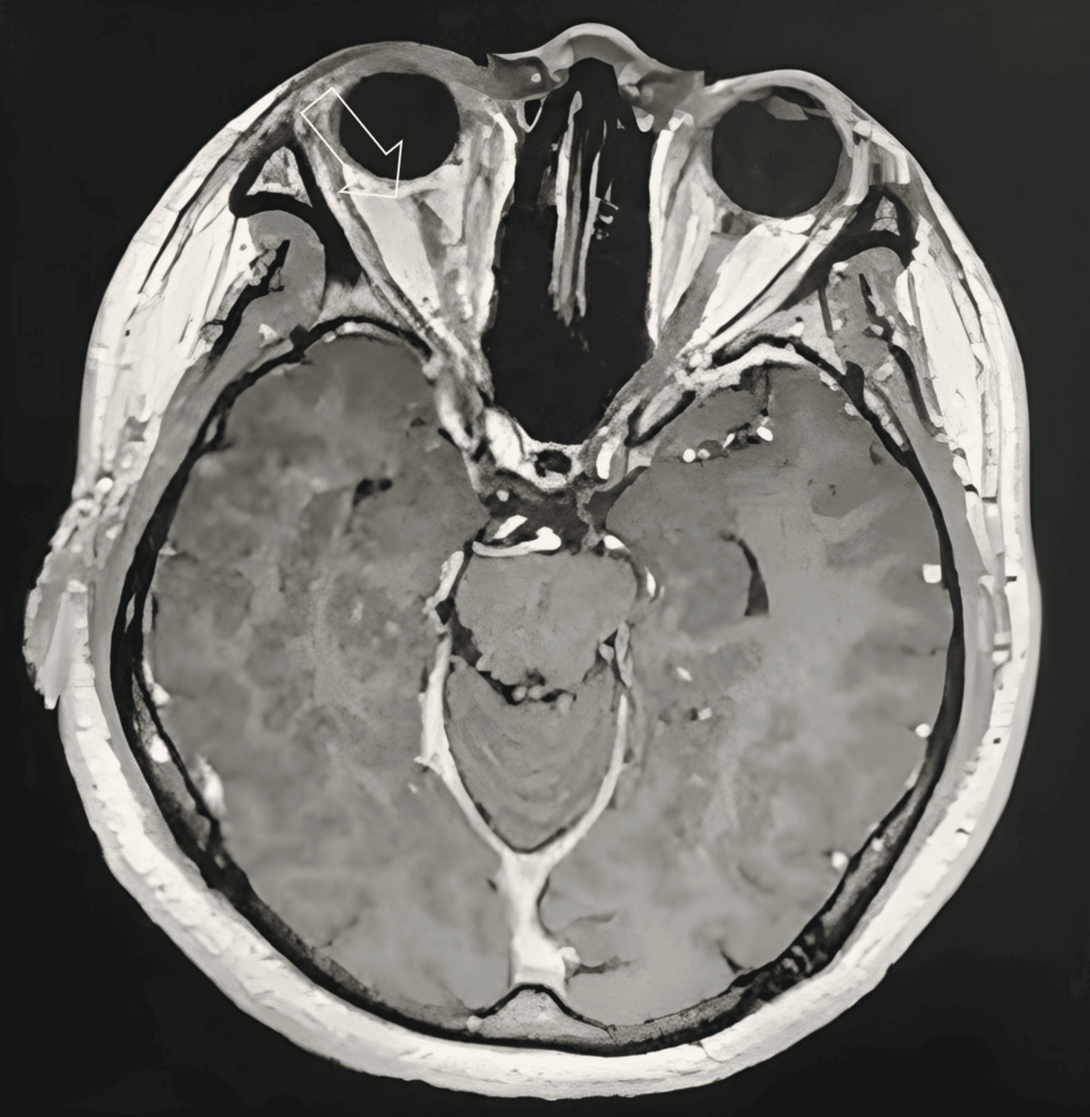
Axial cut MRI of the brain and orbit showing mild heterogenous hyperintensity along the right optic nerve (arrow) with no evidence of central nervous lesion suggestive of demyelinating disease MRI: magnetic resonance imaging

Magnetic resonance imaging (MRI) of the brain was performed and revealed only a mild heterogenous hyperintensity along the right optic nerve, with no additional lesions detected. There were no periventricular, juxtacortical, or infratentorial white matter lesions that are typically associated with demyelinating diseases (Figure 3).

**Figure 4:**
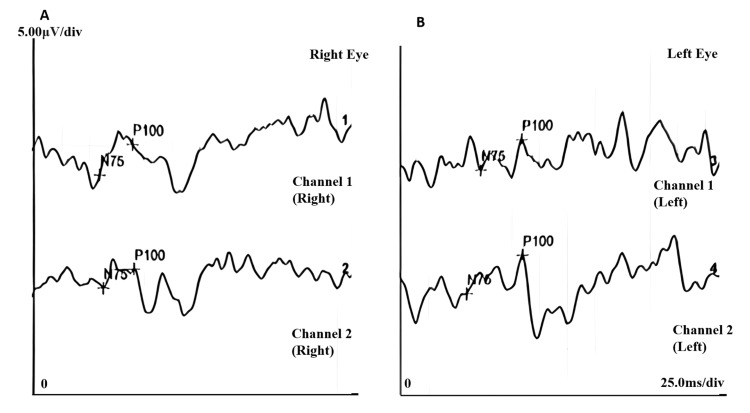
VEP (A) Right eye VEP (channels 1 and 2) showed normal N75 and P100 latencies but a reduced P100 amplitude. (B) Left eye VEP (Channels 1 and 2) showed a prolonged P100 latency with a reduced P100 amplitude. VEP: Visual Evoked Potential

A Visual Evoked Potential (VEP) test was performed, and the findings demonstrated a normal P100 latency in the right eye with reduced amplitude, whereas the left eye showed prolonged latency and reduced amplitude, indicative of impaired conduction along the visual pathway (Figure 4, Table 1). These abnormalities correlate with the clinical findings of optic nerve dysfunction.

**Table 1:** Summary of VEP for both eyes showing reduced amplitude in both eyes, with prolonged P100 latency in the left eye, indicative of optic nerve dysfunction VEP: Visual Evoked Potential

Parameter	Channel	Right eye	Left eye	Reference range	Interpretation
N75 latency (ms)	1	59.9 ms	72.8 ms	78-80 ms	Normal N75 latency in both eyes
	2	63.4 ms	59.9 ms		
P100 latency (ms)	1	89.8 ms	109.8 ms	90-105 ms	Normal P100 latency in the right eye, prolonged P100 latency in the left eye
	2	91.6 ms	110.4 ms		
P100 amplitude (µV)	1	2.33 µV	2.30 µV	3.0-10.0 µV	Reduce P100 amplitude in both the right eye and left eye
	2	1.45 µV	2.92 µV		

Further family history was obtained. He is the second of three children and the only son in the family, with both sisters being healthy. His mother, who was adopted and had no siblings, passed away due to cervical cancer, while his father died from complications related to diabetes mellitus. Socially, he is a non-smoker and consumes alcohol occasionally.

In view of a young man with sequential progressive vision loss with negative inflammatory and demyelinating disease markers, a blood investigation for LHON genetic testing was sent, and a confirmatory result of mitochondrial DNA (mtDNA) G11778A pathogenic mutation was detected.

## Discussion

Leber hereditary optic neuropathy (LHON) is a rare condition, and lack of exposure to this disease can make doctors suspect and treat for other causes of vision loss. LHON is frequently misdiagnosed as optic neuritis due to visual acuity at onset, age at presentation, and pseudo-optic disc edema. LHON primarily affects central vision, progressively resulting in large bilateral centrocecal scotomas. By six months post-onset, optic atrophy becomes apparent, and vision loss tends to stabilize. The chronic phase typically sets in within a year. In rare instances, significant visual recovery can occur, with the m.14484T>C mutation carrying the most favorable prognosis among the three primary mutations [6]. In contrast to LHON, typical optic neuritis (ON) is characterized by acute monocular vision loss accompanied by retro-orbital pain, especially with extraocular movements. It is commonly associated with an underlying demyelinating lesion, such as those seen in multiple sclerosis (MS) [7].

Other than clinical symptoms at presentation, distinctive imaging findings aid in differentiating LHON from optic neuritis. In LHON, fluorescein angiography (FA) shows circumpapillary telangiectatic vessels without dye leakage from the optic disc. In contrast, optic neuritis with disc edema (papillitis) is characterized by inflammation at the optic nerve head, causing vascular leakage and progressive hyperfluorescence of the optic disc [6,7].

Fundus abnormalities in LHON can be further assessed and quantified using optical coherence tomography (OCT). During the acute phase, retinal nerve fiber layer (RNFL) thickening occurs initially in the temporal and inferior quadrants, followed by the superior and nasal quadrants. This pattern aligns with the early involvement of the papillomacular bundle of retinal ganglion cells (RGCs). The thickening of the RNFL results from axonal swelling due to mitochondrial dysfunction. Over time, as optic atrophy sets in, all quadrants exhibit thinning compared to the normal population [8].

Although RNFL changes are observed in both LHON and optic neuritis, their progression differs. In LHON, RNFL thickening is primarily due to mitochondrial dysfunction and axonal swelling, while in ON, inflammation and demyelination drive early RNFL swelling, particularly in the peripapillary region. Additionally, RNFL thinning in LHON follows a more predictable and progressive pattern, whereas in ON, some patients may experience partial recovery, depending on remyelination and axonal regeneration. Optical coherence tomography (OCT) can therefore serve as a valuable tool to distinguish between these two conditions [9-16].

The visual prognosis in LHON is generally poor, with most individuals suffering permanent vision loss and legal blindness [9]. Currently, no definitive treatment exists, although antioxidants such as vitamins B12 and C and coenzyme Q10 may help reduce oxidative stress. Avoiding neurotoxins such as tobacco and alcohol is also recommended. Idebenone has shown some promise in trials but remains limited [9]. Management is primarily supportive, focusing on visual aids, occupational rehabilitation, and social services [11]. Genetic counseling and testing should be offered to affected families to clarify inheritance risks and disease understanding [16].

## Conclusions

In conclusion, LHON should be suspected in young men with recurrent or atypical optic neuritis, especially when MRI is normal, steroids are ineffective, and genetic risk factors are present, to avoid unnecessary treatment and for guiding personalized treatment approaches and improving long-term visual outcomes for affected patients.
